# Spin echo based cardiac diffusion imaging at 7T: An ex vivo study of the porcine heart at 7T and 3T

**DOI:** 10.1371/journal.pone.0213994

**Published:** 2019-03-25

**Authors:** David Lohr, Maxim Terekhov, Andreas Max Weng, Anja Schroeder, Heike Walles, Laura Maria Schreiber

**Affiliations:** 1 Chair of Cellular and Molecular Imaging, Comprehensive Heart Failure Center (CHFC), University Hospital Wuerzburg, Wuerzburg, Germany; 2 Department of Diagnostic and Interventional Radiology, University of Wuerzburg, Wuerzburg, Germany; 3 Chair Tissue Engineering and Regenerative Medicine (TERM), University Hospital Wuerzburg, Wuerzburg, Germany; 4 Translational Center Regenerative Therapies (TLC-RT), Fraunhofer Institute for Silicate Research (ISC), Wuerzburg, Germany; Universitatsklinikum Wurzburg, GERMANY

## Abstract

Purpose of this work was to assess feasibility of cardiac diffusion tensor imaging (cDTI) at 7 T in a set of healthy, unfixed, porcine hearts using various parallel imaging acceleration factors and to compare SNR and derived cDTI metrics to a reference measured at 3 T. Magnetic resonance imaging was performed on 7T and 3T whole body systems using a spin echo diffusion encoding sequence with echo planar imaging readout. Five reference (b = 0 s/mm^2^) images and 30 diffusion directions (b = 700 s/mm^2^) were acquired at both 7 T and 3 T using a GRAPPA acceleration factor R = 1. Scans at 7 T were repeated using R = 2, R = 3, and R = 4. SNR evaluation was based on 30 reference (b = 0 s/mm^2^) images of 30 slices of the left ventricle and cardiac DTI metrics were compared within AHA segmentation. The number of hearts scanned at 7 T and 3 T was n = 11. No statistically significant differences were found for evaluated helix angle, secondary eigenvector angle, fractional anisotropy and apparent diffusion coefficient at the different field strengths, given sufficiently high SNR and geometrically undistorted images. R≥3 was needed to reduce susceptibility induced geometric distortions to an acceptable amount. On average SNR in myocardium of the left ventricle was increased from 29±3 to 44±6 in the reference image (b = 0 s/mm^2^) when switching from 3 T to 7 T. Our study demonstrates that high resolution, ex vivo cDTI is feasible at 7 T using commercial hardware.

## Introduction

Mechanical [1] and electrical [2] properties of the heart are linked to the myocardial micro-architecture, which exhibits alterations in a broad range of cardiovascular pathologies. In the past years cardiac diffusion imaging, especially cardiac DTI (cDTI), has been established as a nondestructive and non-invasive method for analysis of the microstructure of myocardial tissue. Helical configuration of myofiber bundles [3–6] and sheet [7–9] formation of connecting and branching myocytes have been described in ex vivo studies and shown to have high consistency to histological correlation, enabling this method to expand our knowledge of the microstructural basis and progression of cardiovascular diseases.

While technical and methodical advances in cDTI gave rise to in vivo applications [10–13], scan time remains a limiting factor for both angular and spatial resolution. Additionally, DTI is intrinsically limited by signal-to-noise-ratio (SNR) [14–17] and derived metrics are affected by partial volume effects. Thus, ex vivo studies, where scan times are unrestricted and no motion and flow factors exist, remain an important research and validation tool in cDTI applications [18–21].

MRI at ultra-high field strengths (≥7 T) may provide improved SNR, allowing for improvement of the spatial and/or angular resolution, higher b-values, better image quality as well as consistency of diffusion metrics. While there are demonstrations [22, 23] of DTI benefitting from the 7T field in neurological applications, it remains unclear if the SNR advantage will outweigh influences of shortened T_2_, T_2_*, and increased B_0_ and B_1_ inhomogeneity in DTI of the heart.

To date in vivo DTI studies of porcine or human hearts have been limited to field strength of 1.5 T or 3 T, where, due to limited SNR, multiple averages [24–28] are required to ensure consistent results.

Ex vivo studies so far use fixed myocardial tissue to enable the long scan times and/or multiple experiments with parameter variations. In order to produce diffusion data, which is easily reproducible and comparable, we minimized additional tissue treatment and therefore omitted tissue fixation, which shortens the T_2_ relaxation time, a critical parameter in diffusion measurements at ultra-high field strengths.

The main aim of this study was to assess feasibility of cDTI using commercial apparatus and software in a set of healthy, unfixed, porcine hearts at 7 T shortly after excision and to compare derived diffusion metrics to a reference data set measured at 3 T. This will allow the comparison of data consistency between cDTI at 3 T and 7 T. Secondary aim of this study was to assess if SNR loss due to increasing acceleration factors can be sufficiently compensated for by the higher field strength. The analysis of consistency of diffusion metrics, susceptibility induced distortions, and SNR derived from scans with various parallel imaging factors and echo times should further allow adaptation and adjustment of established 3T cDTI techniques to the usage at ultra-high fields in vivo.

## Materials and methods

### Study protocol

Hearts were collected in cooperation with the Translational Center Regenerative Therapies. Animal experiments were approved (reference number: 55.2 2532-2-256) by the District Government of Lower Franconia and the local animal welfare committee and performed according to the German Animal Welfare Act and the EU Directive 2010/63/EU. Male German Landrace piglets, all obtained from the same breeder, and with body weights between 19 and 23.5 kg, were used. More detailed information on the study animals is listed in the S1 Table.

The ratio of heart weight to body weight in 20-30kg pigs (5g/kg) is identical to that of adult humans [29]. The left chambers in porcine hearts are more dominant than in the human heart and the left ventricular wall is much thicker. The interventricular septum in porcine hearts is located more to the right of the heart, while the interventricular septum in the human heart is located in a more central position. This means that the porcine apex is only composed of left ventricular musculature.

For euthanasia, piglets were sedated with azaperone (Stresnil, 2–6 mg/kg) and anesthetized with ketamine (Ursotamin 20–25 mg/kg) intramuscularly in the neck muscle caudal to the base of the ear, before T61 (Embutramid, Mebezoniumiodid, Tetracainhydrochlorid, 0.3–0.4 ml/kg) was administered intravenously via the marginal ear vein.

Immediately after euthanasia the hearts were collected, rinsed and stored in physiological saline solution. They were not fixed in Formalin or other substances. Hearts were centered in plastic containers filled with saline solution using surgical threads. MRI was performed at bore temperature (~19°) on 7T and 3T whole body MRI systems (Siemens MAGNETOM Terra and Prisma, respectively, Erlangen, Germany) within 10 hours after euthanasia using a 1Tx/32Rx head coil. All measurements were therefore performed in a state of rigor mortis. On measurement days we received two hearts, which were excised in one setting. Data for the two hearts was acquired in consecutive measurements in five out of six cases (n = 11). Sample stability measurements were done on separate occasions, since they blocked the scanner for a duration of 12 hours.

### Sample stability

Tissue-stability measurements were made to monitor possible changes in tissue structure, which may occur within the time period between scans at 7 T and 3 T. For this purpose, a mid-cavity slice was imaged continuously in two hearts over a period of 12 hours. The delay between the last excision and the start of imaging was roughly 45 minutes. Myocardial T_2_* and T_1_ values as well as diffusion parameters, such as FA and ADC were measured interleaved in an uninterrupted cycle.

T_2_* was evaluated based on a 2D gradient multi-echo sequence with the following imaging parameters: slice thickness: 5 mm, matrix size: 68 × 176, field of view (FOV): 131 mm × 176 mm, number of averages: 6, TR: 150 ms. Nine echoes per excitation were acquired with TE values between 2.07 ms and 18 ms.

T_1_ was evaluated using the DESPOT1 method [30] with the flip angles: 15°, 30°, and 45°. Ten averages were acquired with TE: 3.69 ms. A supporting B_1_ map for DESPOT1 was derived from additional double flip angle measurements using 6 averages and TR: 2000 ms. FOV and the matrix size remained the same as listed for T_2_* acquisitions.

Twenty diffusion directions according to Skare [31] (b  =  700 s/mm^2^) and five reference images (b  = 0 s/mm^2^) were acquired using a single refocused spin-echo sequence with Stejskal-Tanner diffusion preparation, EPI readout and a GRAPPA acceleration factor R = 3. Measurement parameters were: slice thickness: 5 mm, TE/TR: 55/3000 ms, matrix size: 100 x 128, FOV: 132x170 mm^2^, bandwidth: 2300 Hz/Pixel (readout) and 1299 Hz/Pixel (phase-encode).

### DTI acquisition

At 7 T: Prior to measurements we applied 3^rd^ order shims for a volume covering the whole organ. Whole heart diffusion data sets (n = 11) were acquired with an isotropic resolution of 1.3x1.3x1.3 mm^3^ using a Stejskal-Tanner diffusion preparation and EPI readout (described above). Further parameters were TE/TR: 55/15000 ms, 65 interleaved slices (no gaps), bandwidth: 1302 Hz/Pixel (readout) and 1000 Hz/Pixel (phase-encode), non-accelerated (R = 1) echo train length: 55, matrix size: 84x128, FOV: 111x170 mm^2^ and 5/8 partial-Fourier. 30 diffusion directions (b  =  700 s/mm^2^) [31] and 5 reference (b = 0 s/mm^2^) images were acquired in a total scan time of nine minutes. Measurements with the parameters above were originally chosen for measurements at 3 T [32] and were used here as a first point for comparison between scans at 7 T and 3 T. Measurements with these parameters will be referred to as the reference scan at 7 T (R = 1).

The scan was repeated with GRAPPA acceleration factors R = 2 (n = 7), R = 3 (n = 11) and R = 4 (n = 11), 3/4 partial-Fourier, and an increased readout bandwidth of 2300 Hz/Pixel resulting in minimal TEs and echo train lengths of 50/47/43 ms and 33/21/ 16, respectively. The bandwidth in phase-encode direction was 1299 Hz/Pixel. Total measurement time for the 7T scans was ~65 minutes.

AT 3 T: Whole heart (n = 11) diffusion data sets were acquired at 3T. Reference scan parameters described above were used for image acquisition. The parameters were chosen to optimize for a high isotropic resolution and minimal susceptibility induced distortions in diffusion weighted images at 3 T, while maintaining SNR>25db, which is considered to be in the clinical SNR regime [15].

### DTI data analysis

All Processing was based on images using the vendor reconstruction pipeline. For whole heart scans, motion correction was applied to account for eddy-current induced geometrical distortions. Tensor reconstruction using DSI Studio [33, 34] was achieved as described in [35]. For the Stejskal-Tanner sequence and isotropic samples the observed signal intensity *S*, following diffusion weighting, is:

$$S=S_{0}e^{-bg^{t}Dg}=S_{0}e^{-b\sum_{i,j=x,y,z}\left(g_{i}g_{j}\right)D_{ij}}$$ (1)

where *S*_*0*_ corresponds to the observed signal without diffusion weighting, *g* to the normalized diffusion gradient directions, *b* to the diffusion weighting factor, which, for rectangular gradients, is defined by:

$$b=\gamma^{2}\delta^{2}G^{2}\left(\Delta -\frac{\delta }{3}\right)$$ (2)

and *D* to a 3x3 diffusion Tensor (laboratory frame):

$$D=\left[\begin{array}{ccc} D_{xx} & D_{xy} & D_{xz}\\ D_{yx} & D_{yy} & D_{yz}\\ D_{zx} & D_{zy} & D_{zz} \end{array}\right]$$ (3)

In the definition of the diffusion weighting factor, *γ* corresponds to the gyromagnetic ratio, *δ* and *G* to the duration and amplitude of the applied diffusion gradient in a given direction, and *Δ* to the separation between the applied diffusion gradients. With the introduction of the following two vectors:

$$\bar{D}={\left[D_{xx}\;D_{yy}\;D_{zz}\;D_{xy}\;D_{xz}\;D_{yz}\right]}^{T}$$ (4)

$$\bar{g}={\left[g_{x}^{2}\;g_{y}^{2}\;g_{z}^{2}\;2g_{x}g_{y}\;2g_{x}g_{z}\;2g_{y}g_{z}\right]}^{T}$$ (5)

Eq (1) can be rewritten as:

$$\underset{i,j=x,y,z}{\sum }\left(g_{i}g_{j}\right)D_{ij}={\bar{g}}^{t}\cdot \bar{D}=ln\frac{\left(S/S_{0}\right)}{b}$$ (6)

Since the tensor is symmetric (*D*_*ij*_ = *D*_*ji*_), it can be calculated based on the acquisition of ≥7 images (six or more diffusion weighted images *S*_*k*_ using the diffusion gradients *g*_*k*_ and one reference *S*_*0*_). The resulting system of equations:

$${\bar{g}}_{k}^{t}\cdot \bar{D}=ln\frac{\left(S_{k}/S_{0}\right)}{b_{k}}\;\left(k=1,\ldots ,K;K\geq 6\right)$$ (7)

is solved in matrix form:

$$A\bar{D}=B$$ (8)

where A is a K x 6 matrix:

$$A=[\begin{array}{c} {\bar{g}}_{1}^{t}\\ \vdots \\ {\bar{g}}_{K}^{t} \end{array}]=[\begin{array}{cccccc} g_{1x}^{2} & g_{1y}^{2} & g_{1z}^{2} & 2g_{1x}g_{1y} & 2g_{1x}g_{1y} & 2g_{1y}g_{1z}\\ \vdots & \vdots & \vdots & \vdots & \vdots & \vdots \\ g_{Kx}^{2} & g_{Ky}^{2} & g_{Kz}^{2} & 2g_{Kx}g_{Ky} & 2g_{Kx}g_{Kz} & 2g_{Ky}g_{Kz} \end{array}]$$ (9)

and B a K-dimensional vector:

$$B={\left[\begin{array}{ccc} ln\frac{\left(S_{1}/S_{0}\right)}{b_{1}} & \ldots & ln\frac{\left(S_{K}/S_{0}\right)}{b_{K}} \end{array}\right]}^{T}$$ (10)

The solution is found using the pseudo-inverse (A^+^) of the matrix A:

$$\bar{D}=A^{+}B={\left(A^{T}A\right)}^{-1}A^{T}B$$ (11)

The calculated tensor was used for visualization of fiber bundle tracts, eigenvalue analysis on a voxel-by-voxel-basis and the calculation of fractional anisotropy (FA) and the apparent diffusion coefficient (ADC) using:

$$FA=\frac{\sqrt{3}}{\sqrt{2}}\frac{\sqrt{{\left(\lambda_{1}-\lambda \right)}^{2}+{\left(\lambda_{2}-\lambda \right)}^{2}+{\left(\lambda_{3}-\lambda \right)}^{2}}}{\sqrt{\lambda_{1}^{2}+\lambda_{2}^{2}+\lambda_{3}^{2}}}$$ (12)

and

$$ADC=\frac{\lambda_{1}+\lambda_{2}+\lambda_{3}}{3}$$ (13)

**Fig 1:**
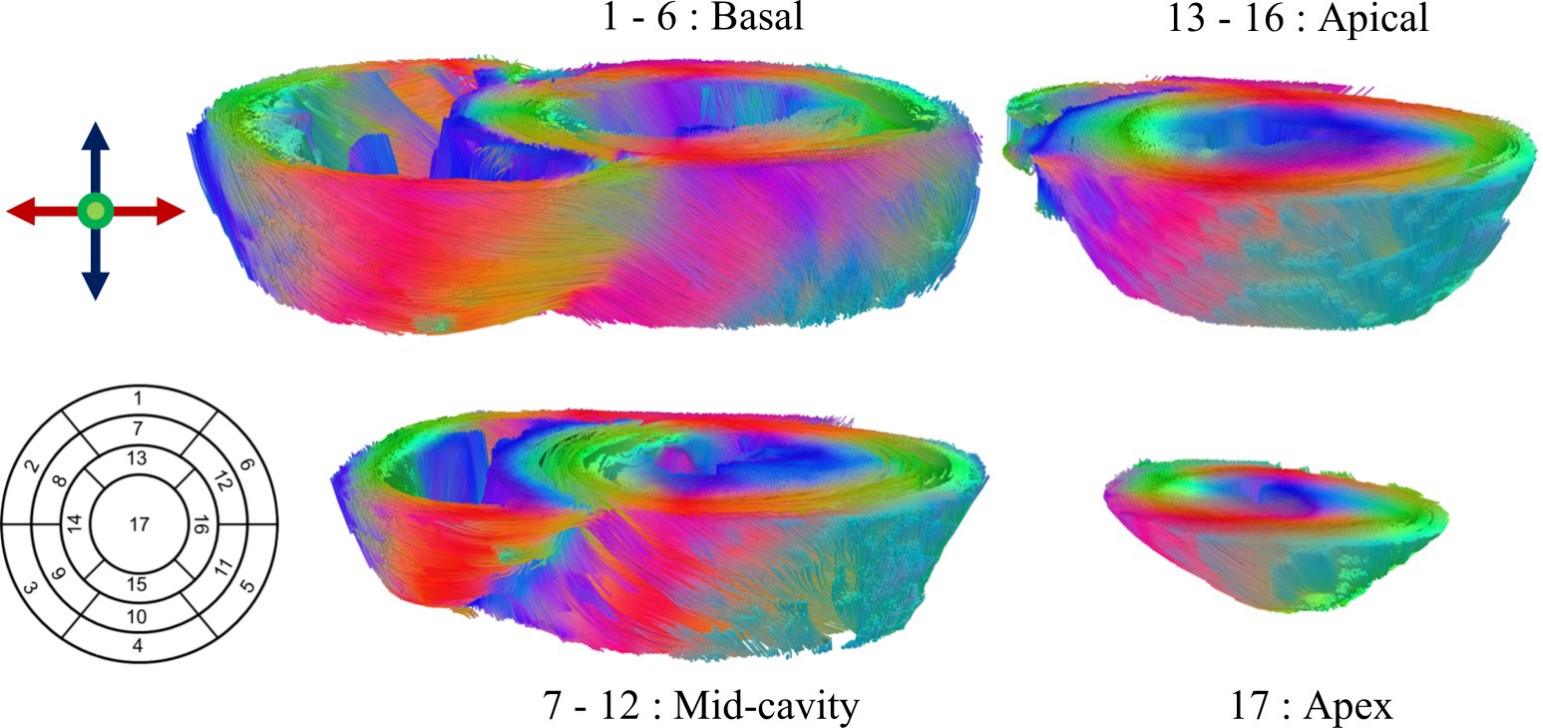
Orientation of the main eigenvector of the diffusion tensor within apex, apical, mid-cavity, and basal parts. Values were calculated from cDTI data measured at 7T and mapped onto fiber tractography of the left and right ventricle. Segmentation was done according to the 17 segment AHA model.

**Fig 2:**
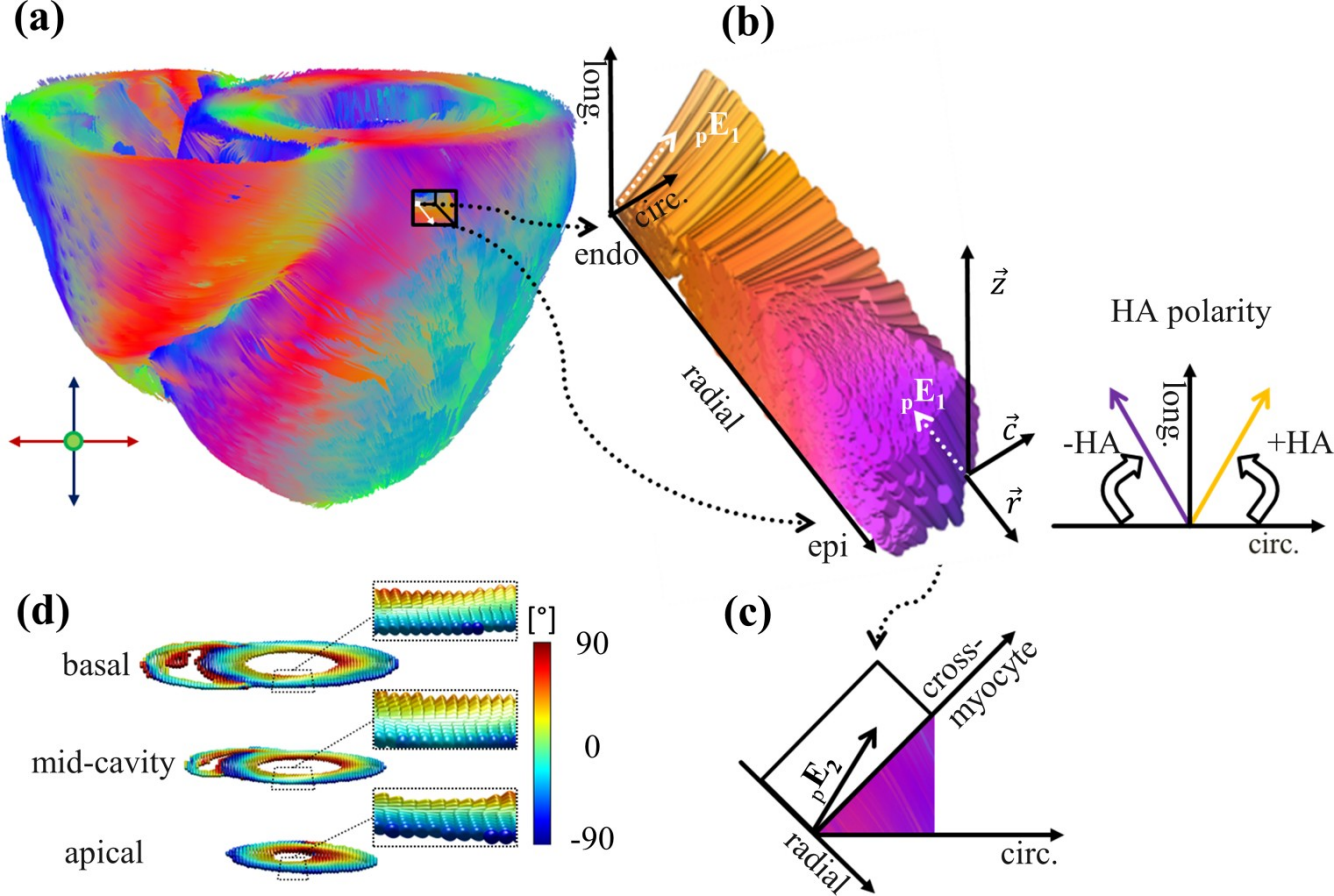
Orientation of primary and secondary eigenvector of diffusion and derived metrics helix angle and E2A. (a) Main eigenvector of diffusion mapped onto fiber tractography of the left and right ventricle. (b) Cut transmural block of reconstructed fibers from a) used to illustrate the parameter helix angle and assignment of its polarity within the local coordinate system of longitudinal $\vec{z}$, radial $\vec{r}$ and circumferential $\vec{c}$ axes. (c) Projected secondary eigenvector angle. (d) Tensor visualization as superquadric glyphs in basal, mid-cavity and apical parts of (a). Color coding resembles helix angle values for single voxels. Zoomed areas show the typical smooth progression of positive right handed fibers in the endocardium to negative left handed fibers in the epicardium.

Here, λ_1_, λ_2_, λ_3_ are eigenvalues of the diffusion tensor sorted by size and *λ* their mean value. All other post processing was accomplished using MATLAB (MathWorks, Natick, USA). First, images were converted to NIfTI format and denoised using the local PCA-denoising algorithm described in [36]. Manual segmentation of the whole heart scans was done according to the 17 segment model [37] of the American Heart Association (AHA). Representative distribution of the segments: basal, mid-cavity, apical and apex is displayed in Fig 1. Resulting myocardial contours were applied to all whole heart scans of the same heart. A local orthogonal coordinate system with longitudinal, circumferential, and radial axes was established and the primary eigenvector (E_1_) was projected in the plane given by circumferential and longitudinal vectors. As illustrated in Fig 2A and 2B the angle between the projected eigenvector and the circumferential direction was defined as the primary eigenvector angle or helix angle (HA) [3, 38]. The orientation of the primary eigenvector depends on localization in the myocardium, describing a smooth transmural progressing from positive right-handed angles in the endocardium to negative left-handed angles in the epicardium (Fig 2D). Analysis of this pattern was done using profile lines between the LV center and epicardial voxels. Profiles were averaged for apical, mid-cavity, and basal parts as well as multiple hearts. The stability of the transmural gradient was assessed using the standard deviation of values at the five myocardial layers: endocardial, sub-endocardial, mid-wall, sub-epicardial, and epicardial.

The secondary eigenvector is associated with sheetlet orientation [7, 38]. As shown in Fig 2C, a cross-myocyte plane was calculated perpendicular to E_1_ for every voxel. The secondary eigenvector was projected onto this plane and the angle between projection and cross-myocyte direction defined as secondary eigenvector angle (E2A).

Comparison between the reference scan at 3 T and 7 T, as well as 7T measurements with GRAPPA factors R = 2, R = 3, and R = 4 was done for medians of ADC, FA, and |E2A| using a Wilcoxon test with a significance level of *P*<0.05. This analysis was performed for apical, mid-cavity, basal parts, and the entire left ventricle. Maximal and minimal deviations of ADC [10^−3^ mm^2^/s] and FA within the AHA segments were used to define a bias range for 7T acquisitions relative to the median 3T reference.

Visualization of diffusion in 3D was done using superquadric glyphs [39] as representations of the primary eigenvector. A generalized version of the deterministic fiber tracking algorithm in [33] was used in DSI studio in order to generate fiber visualization from DT-data. After calculation in Matlab, helix angle values were reintegrated back into DSI Studio, in order to generate a color coded mapping of the parameter as local indices onto reconstructed fibers of the right and left ventricle. The same was done for SNR values within myocardial contours. All fiber tracking was based on a ROI, containing the left and right ventricle from base to apex. Segmentation of this ROI was done using the R = 3 scan and copied to other 7T scans, where applicable. Segmentation of the 3T scan was done separately. An anisotropy threshold of 0.1 was used for fiber termination, filtering out all voxels with FA<0.1. The angular threshold was set to 60° and the step size to 0.5 voxels. Only tracks ranging from 10 mm to 300 mm were accepted and initially a total of 100000 tracks was reconstructed.

### SNR analysis

For the assessment of SNR (n = 9) as a function of parallel imaging we acquired 30 b = 0 s/mm^2^ images of 30 slices of the left ventricle (LV) using the reference scan parameters as well as GRAPPA acceleration factors R = 2, R = 3, and R = 4. No partial Fourier technique was used in GRAPPA accelerated scans. Echo train lengths were 55, 41, 27, and 21, respectively. A region of interest (ROI) was drawn for myocardial tissue of the LV in all 30 slices of the acquired b = 0 images. SNR was calculated according to the multiple acquisition method described by Reeder et al. [40]. Multiple acquisitions with identical scan parameters are used to form a pseudo-time dependent data set. The mean ($\bar{x}$) and standard deviation (*σ*) of every voxel (r) can be calculated over “time” (t), allowing the measurement of SNR on a pixel-by-pixel basis:

$$SNR\left(r\right)=\frac{{\bar{x}}_{t}\left(r\right)}{\sigma_{t}\left(r\right)}$$ (14)

Resulting values were averaged for the previously defined myocardial ROI. SNR in scans with R≥2 and increased bandwidth was compensated using the following two factors, in order to normalize GRAPPA accelerated scans:

$$1:\sqrt{\frac{N_{PE}\left(R=1\right)}{N_{PE}\left(R=k\right)}}\;2:\sqrt{\frac{bw\left(R\geq 2\right)}{bw\left(R=1\right)}}$$ (15)

where N_PE_ corresponds to the number of phase encodings in scans using varying parallel imaging factors *k* and *bw* to the bandwidth. No pre-processing, such as denoising, was applied prior to SNR analysis.

## Results

### Sample stability

**Fig 3:**
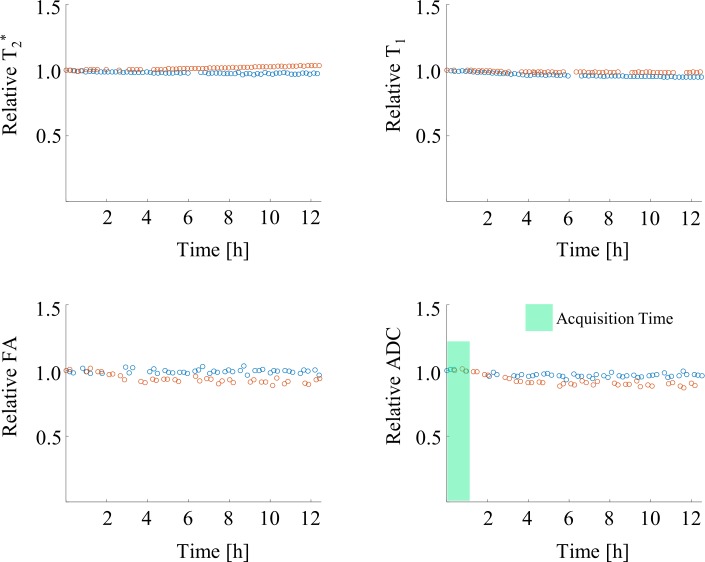
Temporal evolution of sample stability using measures of T2*, T_1_, FA, and ADC. Values are plotted relative to time point t = 0 over a period of 12 hours for two hearts (blue, red). Changes in myocardial T_2_* and T_1_ over this time period for the two hearts were 2–5% and 2–6%, respectively. Mean ± single standard deviation were 0.45±0.01 and 0.45±0.02 for FA and 0.63±0.02 [10^−3^ mm^2^/s] and 0.69±0.03 [10^−3^ mm^2^/s] for ADC respectively. The green area marks the time interval, were the diffusion measurements at 7T would take place. Derived diffusion parameters measured within this time interval were stable.

Experiments monitoring tissue stability over a period of 12 hours showed little sign of change in the measured mid-cavity slice. Time curve developments for relative changes in T_2_*, T_1_, FA, and ADC over the time period of 12 hours are shown in Fig 3. Corresponding changes in absolute values can be found in S1 Fig. Any tissue changes immediately after excision could not be assessed, since scans started approximately 45 min after the second organ excision, due to the transport and preparation time required. No change in LV wall thickness was observed during the measurements. Initial myocardial T_2_* values for the two hearts changed 2% and 5% over the time course of 12 hours, remaining stable with mean ± error of the mean of 20.45 ± 0.15 ms and 20.47 ± 0.28 ms. Relative T_1_ changes over this time period were 2–6%. Mean ± error of the mean for FA and ADC for the two hearts were 0.45±0.01 and 0.45±0.02 and 0.63±0.02 [10^−3^ mm^2^/s] and 0.69±0.03 [10^−3^ mm^2^/s], respectively. ADC values for one heart decreased during the first 4–5 hours of scan time, but remained within measurement precision during typical acquisition times in this study.

### DTI

SNR in b = 0 images of reference scans (R = 1) at 3 T and 7 T was 29±3 and 44±6, respectively. While B_1_ destructive interferences were apparent in saline solution in all 7T acquisitions, such artefacts were mostly not observed within myocardial tissue. Representative images for the various protocols can be found in S2 Fig.

Statistical DTI analysis was performed for 4±1 slices of the apical cap, 10±1 apical, 10±1 mid-cavity, and 11±1 basal slices. A typical segment distribution as well as main eigenvector orientation for fibers of the left and right ventricle reconstructed by tractography is shown in Fig 1.

**Fig 4:**
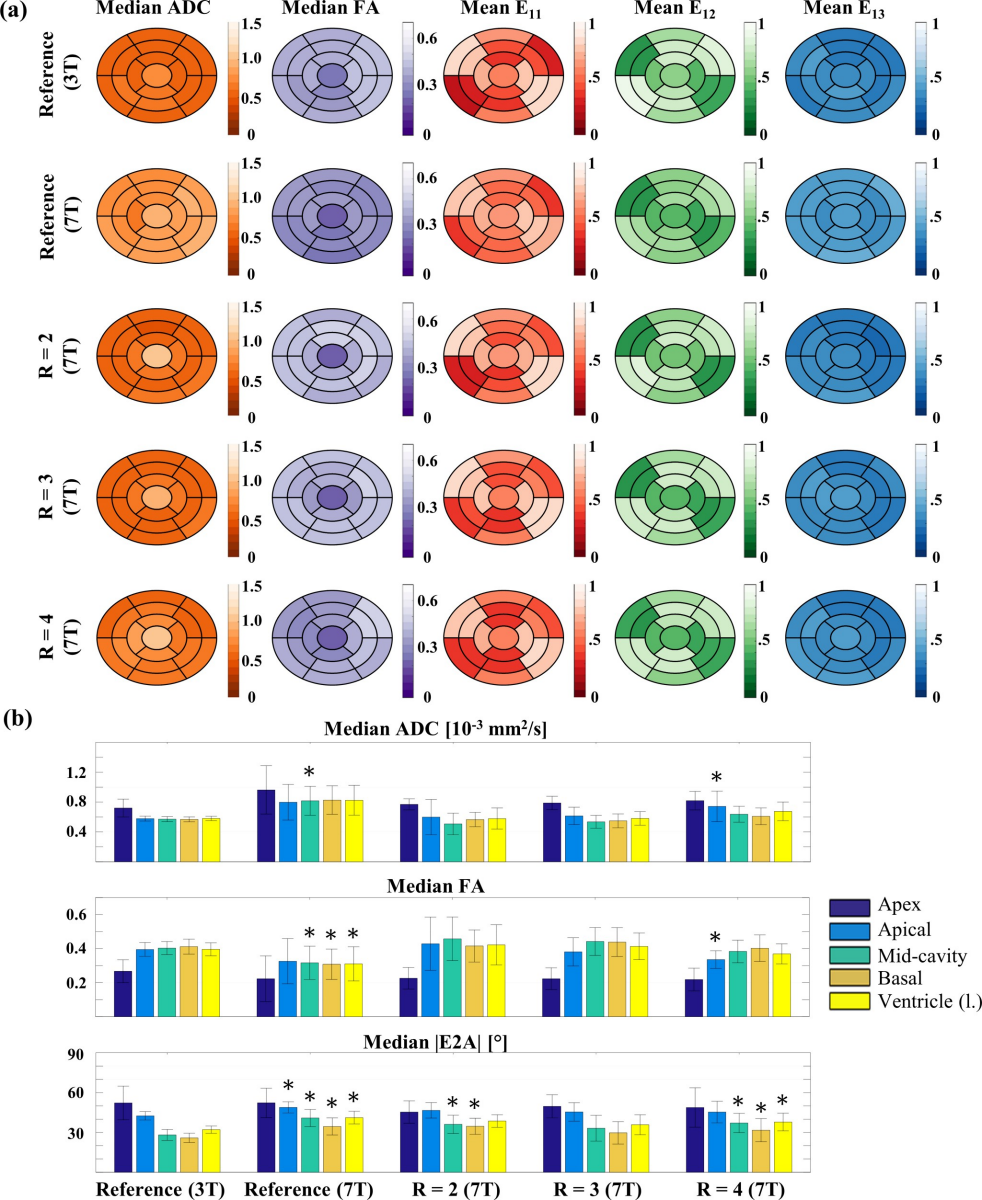
17 segment distributions of diffusion metrics at 3 T and 7 T using varying parallel imaging factors. (a) Median ADC [10^−3^ mm^2^/s], FA, and the three main eigenvector E_1_ components E_11_, E_12_, E_13_ for all 17 segments. Color coding of the vector components corresponds to the “RGB” encoding of spatial orientation for diffusion tensor main axes typically used in DTI (see Fig 1). (b) Values of median ADC, FA and |E2A| averaged for apex, apical, mid-cavity, and basal parts as well as the whole left ventricle A significant difference in Wilcoxon test (*P* <0.05) compared to the 3T reference is indicated by *.

Fig 4A displays median values of diffusion metrics for the 17 segments of the AHA model for all DTI acquisitions. The biggest differences can be found between the reference scan at 3 T and 7 T, particularly for the metrics ADC and FA. Eigenvector orientations appear robust to increasing acceleration at 7 T. The absolute bias ranges of the examined parameters between 3 T and 7 T were as follows: 3–45% (ADC) and 3–26% (FA) using values of the reference at 7 T; 1–12% (ADC) and 1–13% (FA) for GRAPPA factor R = 2; 1–11% (ADC) and 1–12% (FA) for GRAPPA factor R = 3; 1–15% (ADC) and 1–12% (FA) using GRAPPA factor R = 4.

Median ADC, FA, and |E2A| values averaged for apex, apical, mid-cavity, and basal parts as well as the entire left ventricle are displayed in Fig 4B. All metrics measured at 7T exhibit higher standard deviation when compared to the 3T reference. Statistically significant differences in some segments were found for all parameters derived from the 7T R = 1 and R = 4 scans. While calculated metrics from scans using GRAPPA factor R = 2 showed significant differences in the apex for ADC and |E2A| in mid-cavity and basal parts, there were no significant differences for data derived from scans with R = 3.

**Fig 5:**
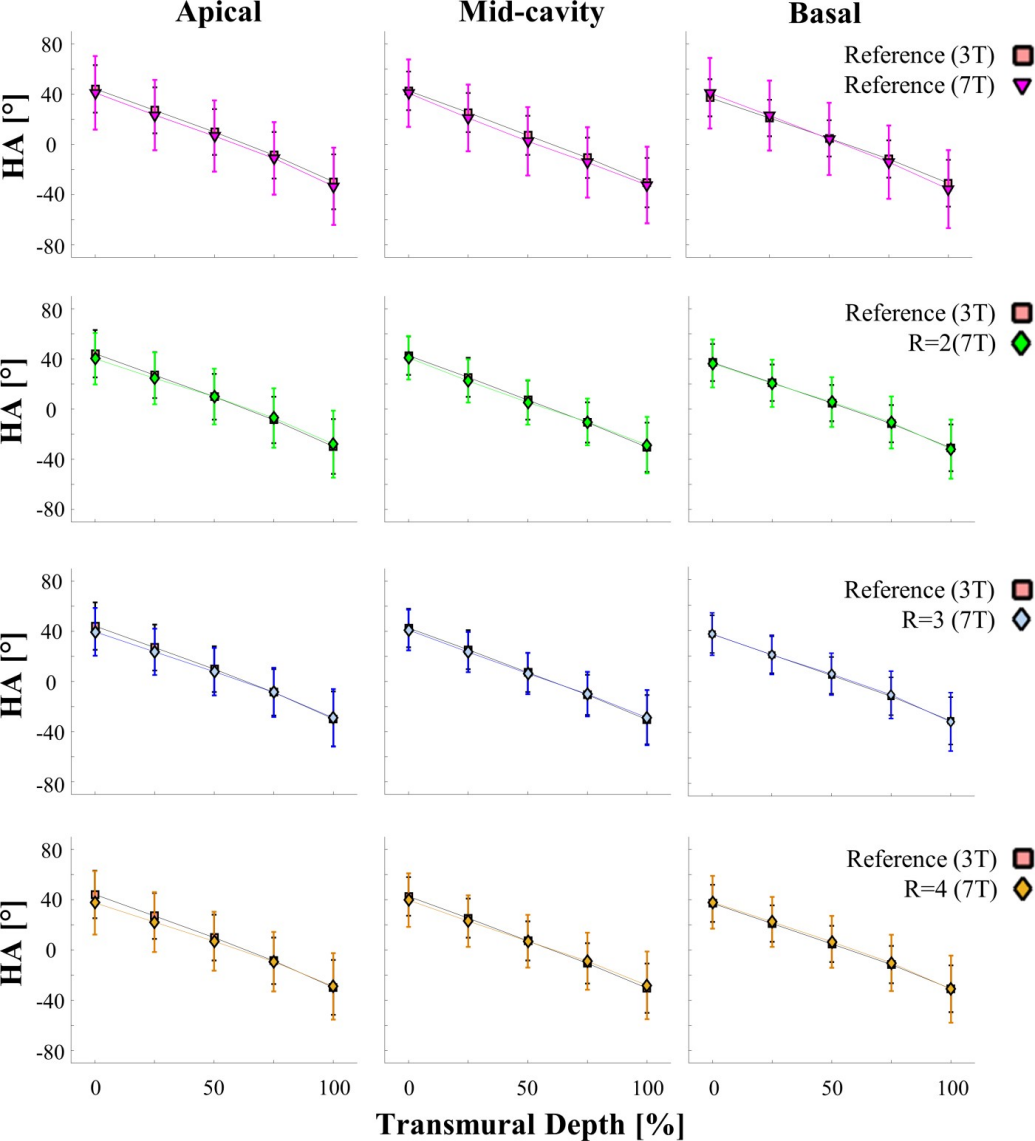
Average transmural helix angle profiles at 7 T and 3 T for apical, mid-cavity, and basal segments. Data was derived from the reference scan at 3 T, the reference scan at 7 T and scans with increasing GRAPPA factors R = 2, R = 3, and R = 4 at 7 T. Data points are displayed for five myocardial layers endocardial, sub-endocardial, mid-wall, sub-epicardial, and epicardial as mean ± one standard deviation. Profiles show helix angle values as a function of transmural depth (%).

Fig 5 shows helix angle profiles averaged for apical, mid-cavity, and basal parts in comparison to the reference (R = 1) data at 3T. Transmural profiles follow the same trend for all acquisitions. Helix angle standard deviations in apical, mid-cavity, and basal segments are displayed in Table 1 for all scans. With 8.7° and 8.6°, averaged over apical, mid-cavity, and basal segments, transmural profiles based on the reference scan at 7 T and scans using R = 4 exhibited the highest variation. Mean values for scans using R = 2 and R = 3 were within a 2° deviation with respect to the 3T reference. GRAPPA factors R>2 lead to an increase in standard deviation relative to R = 2. Compared to the 3T reference, profiles based on the reference scan at 7 T have shown the highest deviation in mean values at different transmural points, particularly in the mid-cavity segment (≤9.1°). The lowest deviation in mean values relative to the 3T reference was found for the scans using R = 2.

**Table 1:** Helix angle standard deviation within apical, mid-cavity and basal parts of the left ventricle.

	Reference (3T)	Reference (7T)	R = 2 (7T)	R = 3 (7T)	R = 4 (7T)
**Apical**	7.3	9.0	5.8	5.9	8.3
**Mid-cavity**	4.4	9.1	6.0	7.0	8.8
**Basal**	3.7	8.0	4.4	5.8	8.6
**Mean**	5.1	8.7	5.4	6.2	8.6

Values were measured at the 5 transmural layers: endocardial, sub-endocardial, mid-wall, sub-epicardial, and epicardial

**Fig 6:**
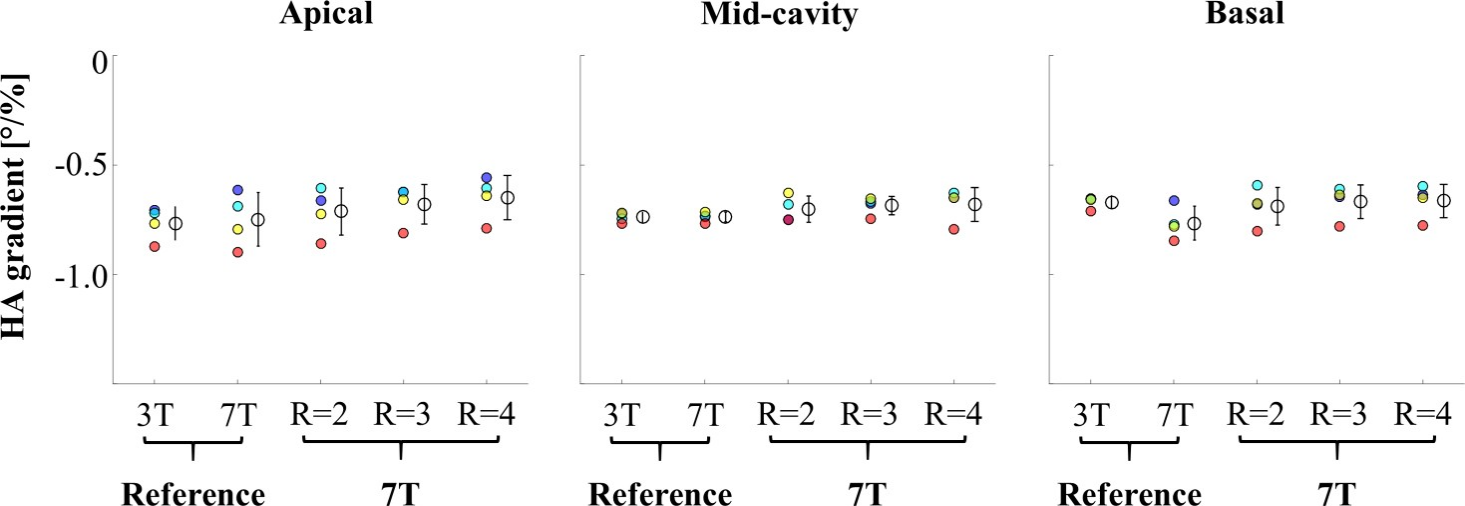
Transmural helix angle gradients between transmural layers. Endocardial, sub-endocardial, mid-wall, sub-epicardial, and epicardial. Gradients were calculated based on the data displayed in Fig 4. Color coding: Endo- to sub-endocardial gradient (blue), sub-endocardial to mid-wall gradient (light blue), mid-wall to sub-epicardial gradient (yellow), and sub-epicardial to epicardial gradient (red). The complete transmural gradient is displayed with mean ± one standard deviation.

Transmural helix angle gradients in degrees per percentage of transmural depth are presented in Fig 6. The highest variation in determined gradient values was found in the apical region. Scans using the reference parameters at 7 T lead to the biggest difference in mean gradients for apical and basal regions, while showing the smallest difference in mid-cavity regions. Averaged over all regions the reference at 3 T and the R = 3 scan exhibited the lowest standard deviations. The smallest difference in mean values in relation to the 3T reference was found for R = 2.

**Fig 7:**
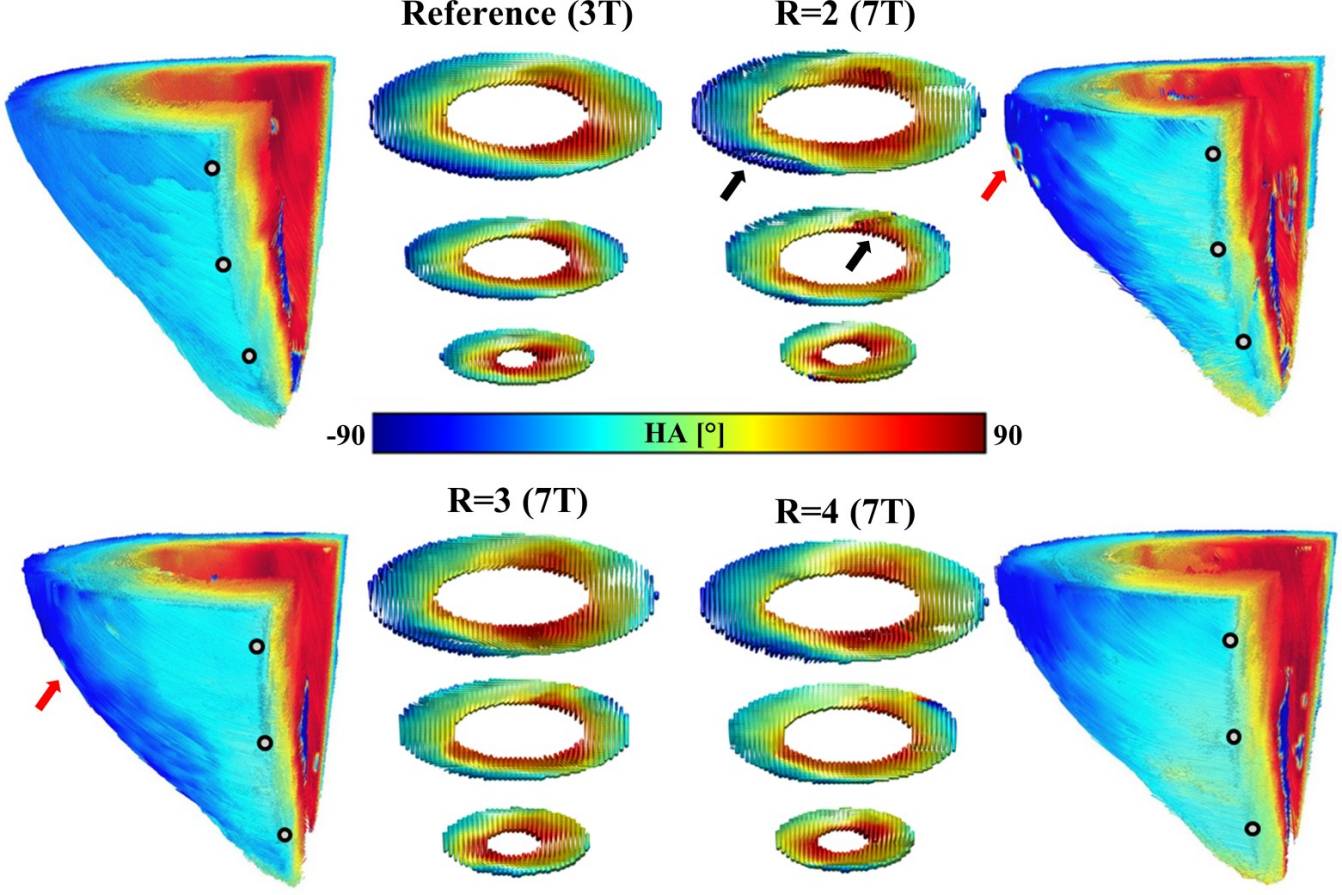
Tractography and helix angle comparison between the 3T reference and GRAPPA accelerated scans at 7 T. Examples of left ventricle tractography from the same heart are displayed for the 3T reference and GRAPPA accelerated scans at 7 T showing differences in resulting tractography and helix angle values. Grey points indicate the position of selected apical, mid-cavity and basal slices. The diffusion tensor within the myocardium of the left ventricle in these slices is visualized as a superquadric glyph. Areas with glyph variation and underlying changes in the diffusion tensor are indicated by a black arrow and areas of helix angle variation in the epicardium with a red arrow.

Fig 7 shows an excerpt of fiber tractography of the left ventricle and the diffusion tensor represented as superquadric glyphs in apical, mid-cavity, and basal slices for the same heart of both the reference scan at 3 T and GRAPPA accelerated scans at 7 T. Helix angle values across the myocardium of reconstructed fibers transition smoothly in the 3T reference and the scan using GRAPPA factor R = 4. Transitions, particularly in the epicardium, are less smooth for the scans using GRAPPA factors R = 2,3 and appear patchy. The highest variation for helix angle values was found for epicardial voxels and areas within the left ventricle involving papillary muscle. In addition there are areas with varying glyph form (black arrows), indicating variations in the underlying diffusion tensor for scans at 7 T. These changes are severe for scans using GRAPPA factor R = 1 and become less severe with increasing acceleration.

### SNR

**Fig 8:**
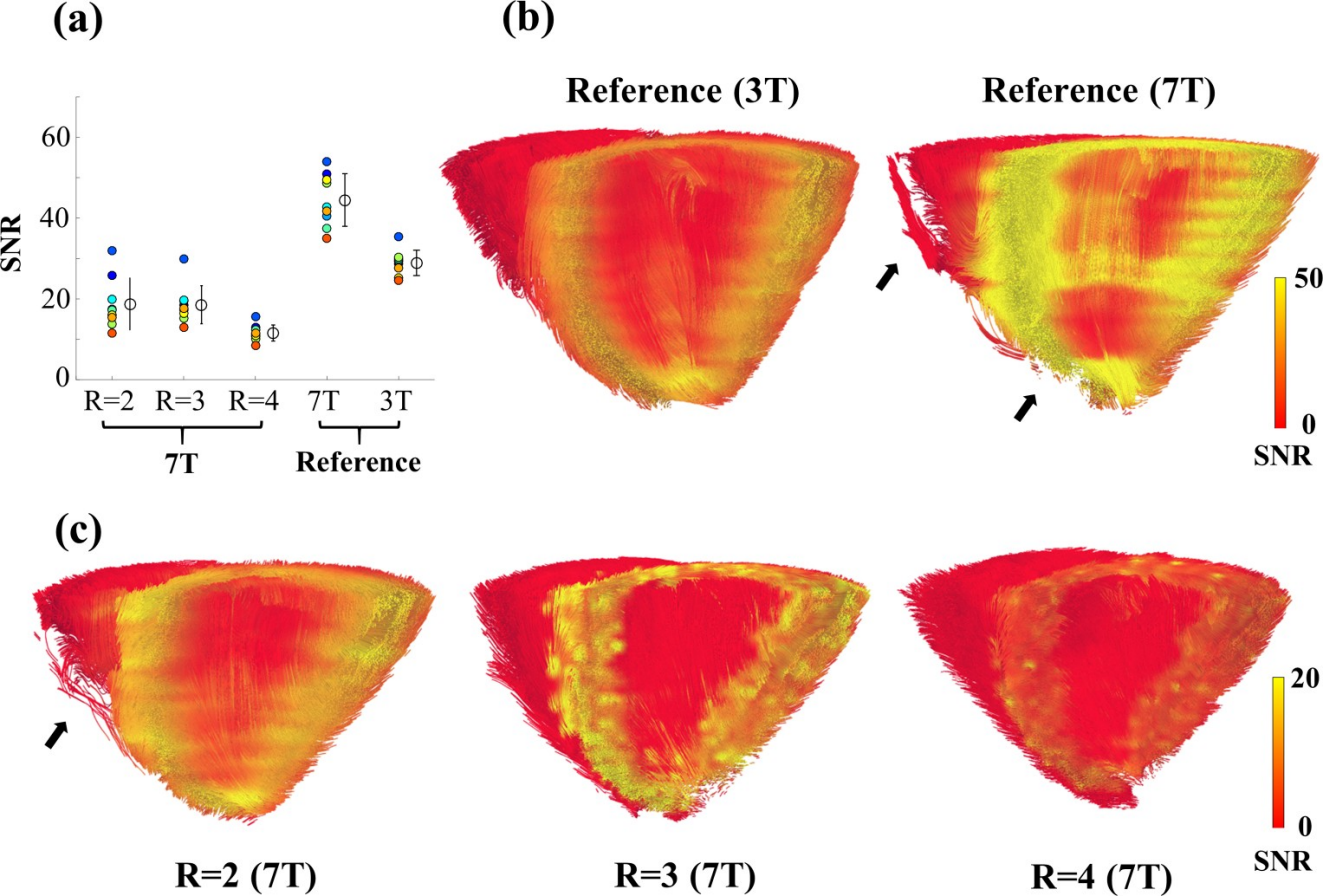
Calculated SNR in the 3T reference, the 7T reference and GRAPPA accelerated scans at 7 T. (a) Shown are average SNR (normalized) values of the hearts measured at 7 T, the reference hearts measured at 3 T and their mean ± single standard deviation. (b) A tractography excerpt of the left and right ventricle from the same heart is displayed for 30 slices of the 3T reference and the 7T reference. Mapped to reconstructed fibers are the measured SNR values. (c) Tractography volumes identical to the 7T reference in (b) are shown for GRAPPA accelerated scans and resulting SNR. Areas of incomplete or diffuse fiber reconstructions are indicated by a black arrow. SNR values were only calculated for myocardial contours of the left ventricle, setting voxels within the right ventricle and papillary muscle to 0. Threshold for tractography was set to a FA value 0.1 for all visualizations.

Fig 8A displays average SNR, normalized using the factors in Eq (15), in myocardial tissue in unaveraged b = 0 s/mm^2^ images acquired for this study. Means of SNR were 29±3, 44±6, 17±6, 19±5, 13±2 for the reference at 3 T, the reference at 7 T, and scans using GRAPPA factors R = 2–4, respectively. Representative, for the same heart, calculated values, mapped to fiber tractography of the 3T and the 7T reference scan are shown in Fig 8B and corresponding tractography for GRAPPA accelerated scans in Fig 8C. Mapped SNR values illustrate the distribution within the myocardium of the left ventricle. The SNR gain at 7 T is clearly visible. Incomplete or diffuse reconstructions in the reference scan at 7 T and the scan using GRAPPA acceleration factor R = 2 are indicated by black arrows.

## Discussion

Our results demonstrate that cDTI in unfixed porcine hearts at 7 T is feasible and can lead to improved SNR in DTI acquisitions of myocardial tissue. Comparison to a reference data set of the same hearts measured at 3 T shows that essential DTI features such as HA, |E2A|, FA, and ADC, do not significantly change with B_0_ field strength, given sufficiently high SNR and geometrically undistorted images. This is an important finding with regard to future studies.

Measurements in vivo will require additional measures to reduce susceptibility in areas close to the lung. This can be accomplished via dedicated shimming methods [41] and/or further reduction of the echo train using reduced field-of-view approaches [13, 42].

Obtained FA values in the unfixed heart using b = 700 s/mm^2^ are 0.41±0.04 at 3 T (R = 1), 0.43±0.07 at 7 T (R = 2) and 0.42±0.06 at 7 T (R = 3). Using a similar b value (b = 800 s/mm^2^) in porcine hearts at 3T, Wu et al. [21] reported FA = 0.32±0.01 and Pashakhanloo et al. [43] FA = 0.37±0.04. Literature values above refer to formalin based tissue fixation prior to DTI measurements. FA discrepancy observed in our study with respect to values cited above are in agreement with studies analyzing the impact of tissue fixation on diffusion metrics. Mazumder et al. [44] reported FA values of 0.42 ± 0.028 in porcine hearts prior to formalin fixation, which dropped to 0.26 ± 0.034 after fixation. A similar observation was made in [45], which reports a fixative concentration dependent decrease in FA compared to unfixed myocardial tissue.

Median ADC values for apical to basal segments in our study range from 0.53–0.62 [10^−3^ mm^2^/s] (3T) and 0.51–0.66 [10^−3^ mm^2^/s] (7T, R = 3). While the corresponding values of 0.671 ± 0.106 and 0.633 ±0.04 [10^−3^ mm^2^/s] reported by Wu and Pashakhanloo et al. in fixed hearts are higher, our results are in good agreement with Mazumder et al., who reported an ADC of 0.52 ± 0.026 [10^−3^ mm^2^/s] in unfixed tissue, and observed an increase in ADC following tissue fixation. In addition to different tissue preparation, the age and breed of the pigs may have an influence on derived ADC and FA values as well. Compared to heart regions in the middle of the ventricle, the number of analyzed voxels in the apex is relatively low. Differences in FA and ADC compared to the other regions may therefore be caused by a mix of structural differences and an increased role of partial volume effects.

ADC, |E2A|, and helix angle values were used for validation versus the 3T reference. Standard deviations were particularly high for the reference scan at 7T, resulting in a significant difference compared to the 3T reference in ADC of mid-cavity segments, despite systematic overestimation of this metric, and multiple significant differences for the metrics FA and |E2A|. DTI measurements using EPI readout without reduction in the number of phase encoding steps, due to parallel imaging acceleration, do not seem feasible at 7T, leading to a strong bias in derived diffusion metrics. While ADC is influenced by low SNR (<20db), there was only one significant difference between GRAPPA accelerated scans at 7T using R = 4 and the 3T reference in apical segments. FA was similarly affected, while main eigenvector orientations appeared robust for GRAPPA accelerated scans. Simulations [46] have shown that FA and parametric angles are largely independent of the trade-off between the number of acquired directions and SNR in b = 0 images. However, the acquisition of 30 diffusion directions leads to an increased robustness of measured FA and decreases the probability of a measured FA bias caused by extreme cases of underlying tissue orientation. In addition to the ADC, |E2A| is also affected by low SNR (<20db). In R = 4 scans this leads to significant differences compared to the reference at 3 T and scans using R = 3. This is in agreement with recent work of Scott et al. [16] demonstrating that noise leads to a loss of both precision and accuracy in derived |E2A| values.

Acceleration of image acquisition using parallel imaging at 7 T is a trade-off between susceptibility effects for long echo trains at low acceleration, SNR loss as acceleration penalty, and an SNR gain for shortened EPI readouts due to lower T_2_* induced losses. Undersampling-effects on the k-space filter, and thus point spread function, were outside the scope of this work and are most likely masked by the applied denoising algorithm. For the tested conditions, we found that GRAPPA acceleration factor R = 3 was optimal for several derived DT-features. Taking the number of phase encoding steps into account, SNR comparison showed, that scans using R = 3 benefitted from the shorter EPI readout leading to an increase in relative SNR compared to R = 2. We observed a significant loss in SNR compared to the reference (R = 1) at 7 T when using R = 2, even when taking into account SNR penalty normalization. Most likely this is due to the relatively small size of the piglet heart in comparison to the size of the coil elements, which leads to an enhanced effect of the g-factor on noise amplification [47, 48] in GRAPPA reconstructed images. Additionally, as shown in [49], SNR decreases for high acceleration factors at ultra-high field strength due to fundamental electromagnetic factors, even considering optimal coil sensitivity profiles. For our setup this effect may already occur for the moderate acceleration factors used. A systematic evaluation of different coil combinations (sum of squares vs adaptive combine) and a comparison of GRAPPA versus SENSE acceleration may be subject of future studies.

The results also show that long echo times and readouts at 7 T lead to susceptibility effects, causing deformation of the reconstructed diffusion tensor. While ADC and FA values are changed significantly, main eigenvector orientation appears to be robust in the presence of susceptibility induced geometrical distortions. This is in agreement with observations made in our previous work [50], where susceptibility effects on the main eigenvector were analyzed at 3 T. It was demonstrated, that, while there are changes in the eigenvector components, the ratio between them stays similar, resulting in small orientation changes of the main eigenvector of diffusion. As shown in Fig 4B changes in the |E2A| are more pronounced, resulting in significant deviations, when compared to our 3T reference. The need for highly parallel imaging reported [22] for DTI in the brain at 7 T, holds true for ex vivo cDTI at 7 T as well.

Despite the observed advantages in SNR, the increased B_0_ (7 T) also increases demands on the measurement setup and introduces additional limitations in comparison to lower field strengths (≤3T). For fixed hearts and certain scan times proton-free and low electrical permittivity synthetic oils such as perfluorpolyether (e.g. Fomblin) can be used to minimize both B_0_ and B_1_ effects of the surrounding on image quality of myocardial tissue, without effecting T_1_, T_2_, and targeted histo-architecture [51]. While the effects of Fomblin on T_2_ and T_2_* in unfixed tissue have been shown to be insignificant [52], diffusion metrics may still vary depending on the sample preparation. The use of Fomblin was therefore omitted in this study. Additional advantages may be gained using B_0_ correction techniques, enabling imaging with lower acceleration factors, and thus maintaining higher SNR. However, in our experience, non-negligible second and third order shim terms are necessary to correct B_0_ field inhomogeneity in the heart at 7 T. Established correction techniques might therefore show weaker performance at ultra-high fields. Achieving stable solutions of optimization-based distortion correction methods is additionally limited by the smaller organ size compared to the brain, which results in smaller numbers of available pixels to solve the minimization problem. Exploration of the limits of existing techniques might be subject of a future ex vivo study, but were outside the scope of this work.

|E2A| has been shown to depend on the heart phase and therefore the contraction state of the organ, which is why hearts are often forced into a set contraction state prior to fixation and following ex vivo analysis of laminar structure. The question whether tissue fixation, which is predominantly used at lower fields for ex vivo DTI, is reasonable for DTI of the porcine heart at 7 T, is still open, and may be subject to future studies. However the reduced $T_{2}^{*}$ due to tissue fixation will further the need for higher parallel imaging factors. The contraction state of hearts in this study was not controlled. The evaluation of |E2A| was therefore exclusively to analyze reproducibility using varying acquisition protocols.

Optimization of different components of the measurement setup may lead to improved robustness and precision of high resolution scans with long acquisition time. For the high acceleration factors (R≥3) required, it is beneficial to use a dedicated multi-channel array, e.g. a 64 element array as used in [53]. The heart should be placed in a dedicated spherical container filled with liquid (magnetic susceptibility and electric permittivity similar to tissue), in order to achieve optimal 3^rd^ order shimming results and minimize SNR degradation due to inhomogeneous B_1_. Prior to measurements the influence of such liquids on DTI metrics should be tested.

Additional advantages in diffusion imaging may be gained using pulse sequence optimization. Long echo times required for diffusion encoding reduce available SNR at 7 T and increase the influence of susceptibility effects. A stimulated echo approach may, despite the factor two disadvantage in SNR compared to the standard spin echo sequence, increase SNR in two ways: 1) Signal during the mixing time, where magnetization is stored in the longitudinal direction, decays with T_1_ time, which is increased at 7 T. 2) The mixing time also contributes to diffusion encoding and thus enables to shorten periods affected by T_2_ relaxation, which is short at 7 T. Both factors become particularly pronounced in in vivo measurements where the mixing time is equal to the RR-interval.

While an increase in field strength can lead to increased SNR, it will also lead to changes in the distribution of field inhomogeneity introduced by locally varying orientations of fiber bundles within the heart. In addition to studies that leverage ultra-high field strengths for resolution [54], this has led to studies exploring high resolution T_2_* imaging [55, 56], quantitative susceptibility mapping, or even measurements of a susceptibility tensor [57] as alternative methods to diffusion imaging. While the feasibility of these methods has been demonstrated for beating, isolated hearts and fixed, ex vivo hearts, there have been no demonstrations in vivo. Macroscopic field inhomogeneity and variations of susceptibility due to changing blood oxygenation levels may limit a direct translation to in vivo applications. Additionally, in order to get sufficient orientation information for the reconstruction of a susceptibility tensor, the specimen needs to be rotated with respect to the magnetic field, which complicates a practical translation to in-vivo measurements.

We obtained DTI data of the fresh hearts at 7 T within 1–3 hours after euthanasia. The 3T MRI system is predominantly clinically used, which limited the time slots for acquisition of our reference data set to 5–10 hours after euthanasia. Usually, 3T and 7T scans should be performed in random order to minimize systematic errors. Considering our results that T_2_*, T_1_, and diffusion metrics are constant over 12 hours, we believe that the time between scans and the missing randomization are no critical factors for the results.

## Conclusions

In this study we demonstrate feasibility of whole heart, high resolution DTI acquisitions of the healthy, unfixed porcine heart at 7 T using commercial hardware. For the coil used, we conclude that a minimum of R = 3 will provide the best compromise between the effects of susceptibility induced distortions and SNR losses. We also conclude that a dedicated coil setup for ex vivo measurements of organs the size of a piglet heart is necessary to enable the use of acceleration factors ≥4.

## Supporting information

S1 Fig Temporal evolution of sample stability using measures of absolute T2*, T_1_, FA, and ADC. Values are plotted relative to time point t = 0 over a period of 12 hours for two hearts (blue, red). The green area marks the time interval, were the diffusion measurements at 7T would take place.
(TIF)

S2 Fig Representative reference and diffusion weighted images for the various acquisition protocols used. Diffusion weighted images #1–4 correspond to the first 4/30 gradient orientations according to Skare (31).
(TIF)

S1 Table Information on study animals and corresponding estimates of left ventricle size and heart weight. Heart size and weight were not measured within this study. Shown values for the size are estimates of the size of the left ventricle based on the number of slices included in the AHA segmentation and the slice thickness during data acquisition. Values for the heart weight are estimates for the total heart weight based on the heart weight to body weight ratio of 5g/kg reported by Lelovas et al (29).
(DOCX)
