# Supplementary figures and images for: Spin echo based cardiac diffusion imaging at 7T: An ex vivo study of the porcine heart at 7T and 3T
Source: PLoS One. 2019 Mar 25;14(3):e0213994. doi: 10.1371/journal.pone.0213994 (PMC6433440; doi:10.1371/journal.pone.0213994)

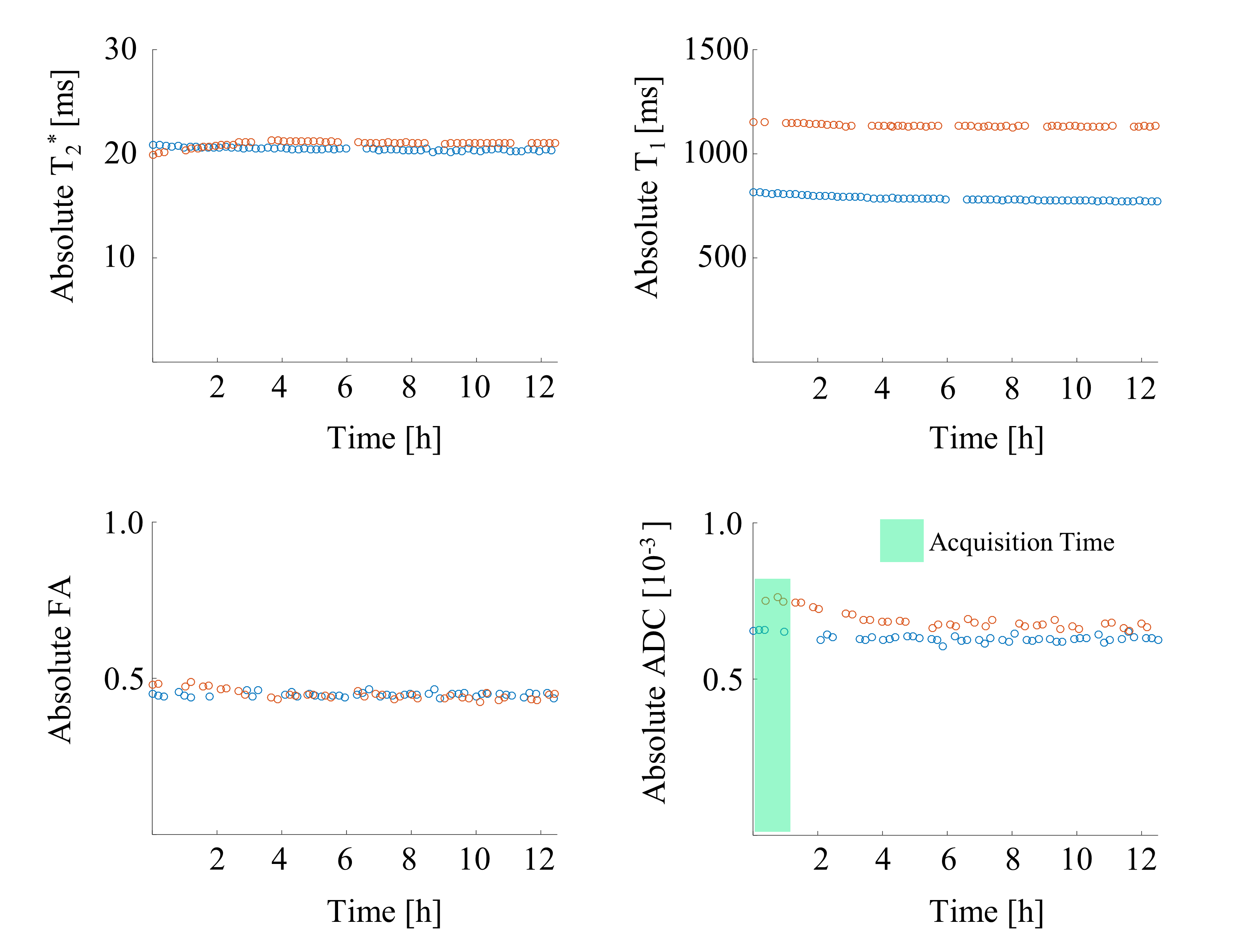

Supplement: S1 Fig — Values are plotted relative to time point t = 0 over a period of 12 hours for two hearts (blue, red). The green area marks the time interval, were the diffusion measurements at 7T would take place. (TIF) [file pone.0213994.s001.tif]

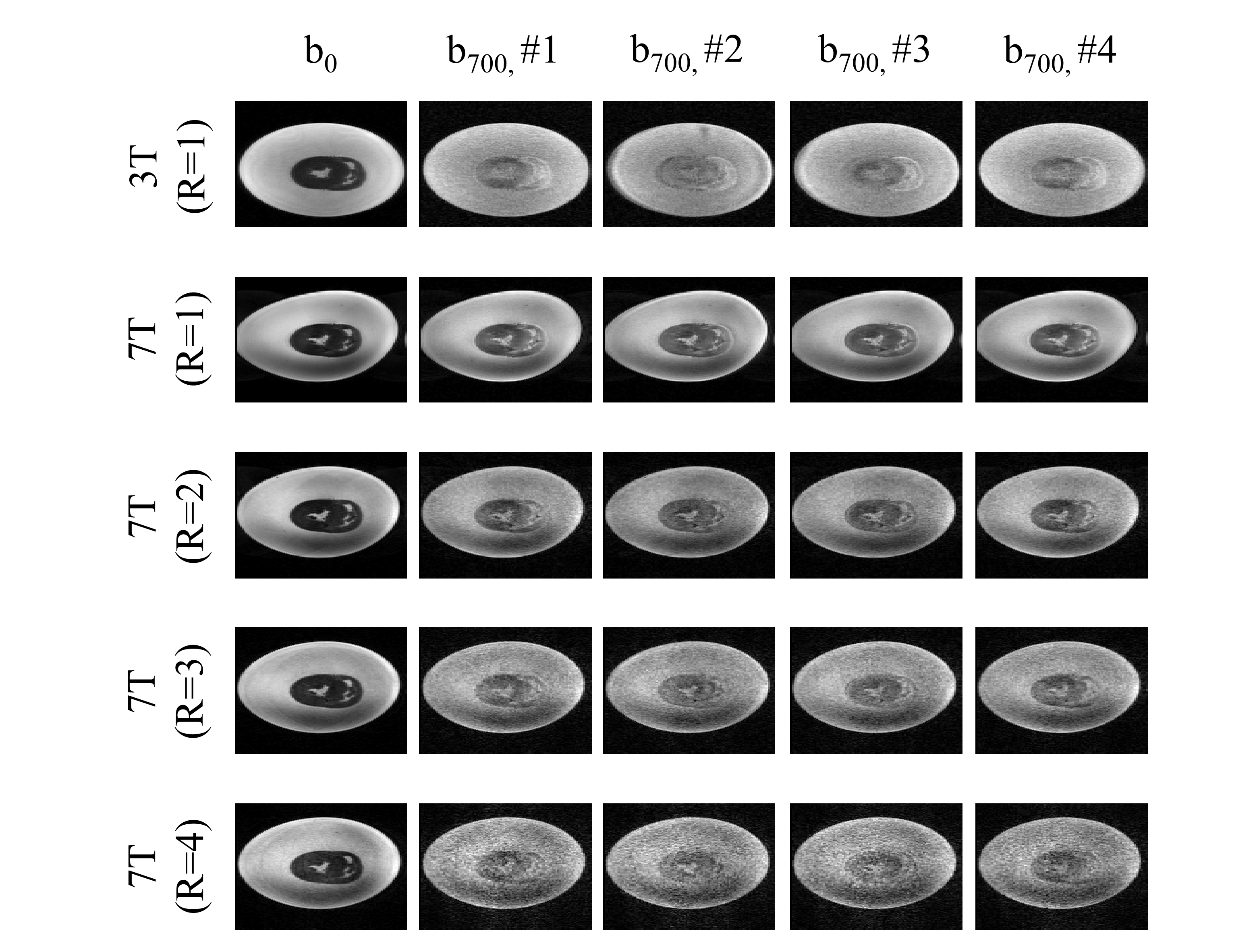

Supplement: S2 Fig — Diffusion weighted images #1–4 correspond to the first 4/30 gradient orientations according to Skare (31). (TIF) [file pone.0213994.s002.tif]
