# Supplementary material for: Spin echo based cardiac diffusion imaging at 7T: An ex vivo study of the porcine heart at 7T and 3T
Source: PLoS One. 2019 Mar 25;14(3):e0213994. doi: 10.1371/journal.pone.0213994 (PMC6433440; doi:10.1371/journal.pone.0213994)
Supplement: S1 Table — Heart size and weight were not measured within this study. Shown values for the size are estimates of the size of the left ventricle based on the number of slices included in the AHA segmentation and the slice thickness during data acquisition. Values for the heart weight are estimates for the total heart weight based on the heart weight to body weight ratio of 5g/kg reported by Lelovas et al (29). (DOCX) [file pone.0213994.s003.docx]

S1 Table. Information on study animals and corresponding estimates of left ventricle size and heart weight.

| **Animal**  **#** | **Age**  **(days)** | **Weight**  **(kg)** | **Size - Base to Apex** | | **Estimated Weight**  **(g)** |
| --- | --- | --- | --- | --- | --- |
|  |  |  | **slices** | **(mm)** |  |
| **1** | 71 | 21.5 | 36 | 46.8 | 107.5 |
| **2** | 71 | 21.5 | 34 | 44.2 | 107.5 |
| **3** | 64 | 20.0 | 36 | 46.8 | 100 |
| **4** | 79 | 21.0 | 38 | 49.4 | 105 |
| **5** | 78 | 21.0 | 36 | 46.8 | 105 |
| **6** | 91 | 21.0 | 31 | 40.3 | 105 |
| **7** | 73 | 22.0 | 33 | 42.9 | 110 |
| **8** | 62 | 22.0 | 42 | 54.6 | 110 |
| **9** | 62 | 23.5 | 40 | 52.0 | 117.5 |
| **10** | 57 | 19.0 | 37 | 48.1 | 95 |
| **11** | 57 | 19.0 | 38 | 49.4 | 95 |
| **mean** | 70 | 21 | 36 | 47.4 | 105 |

Heart size and weight were not measured within this study. Shown values for the size are estimates of the size of the left ventricle based on the number of slices included in the AHA segmentation and the slice thickness during data acquisition. Values for the heart weight are estimates for the total heart weight based on the heart weight to body weight ratio of 5g/kg reported by Lelovas et al (29).
